# Nanostructured Implant–Tissue Interface Assessment Using a Three-Dimensional Gingival Tissue Equivalent

**DOI:** 10.1021/acsomega.4c02253

**Published:** 2024-07-03

**Authors:** Maria Antonia Llopis-Grimalt, Marta Munar-Bestard, Guillem Ramis-Munar, David Smith, Tobias Starborg, Karl E. Kadler, Marta Monjo, Joana M. Ramis

**Affiliations:** † Group of Cell Therapy and Tissue Engineering, Department of Fundamental Biology and Health Sciences, Research Institute of Health Sciences (IUNICS), University of the Balearic Islands, Palma 07122, Spain; ‡ Health Research Institute of the Balearic Islands, IdISBa, Palma 07010, Spain; § Cellomics Unit, Research Institute of Health Sciences (IUNICS), University of the Balearic Islands, Palma 07122, Spain; ∥ Wellcome Centre for Cell-Matrix Research, Faculty of Biology, Medicine and Health, University of Manchester, Michael Smith Building, Oxford Road, Manchester M13 9PT, United Kingdom

## Abstract

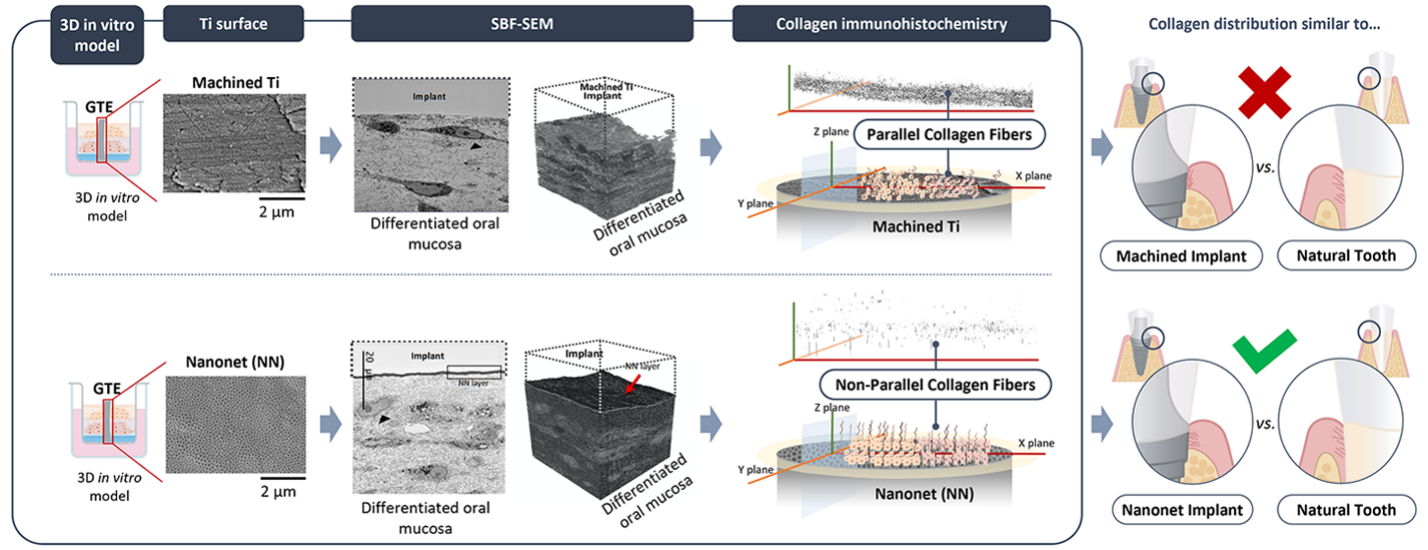

Improved soft tissue integration (STI) around dental implants is key for implant success. The formation of an early and long-lasting transmucosal seal around the implant abutment might help to prevent peri-implantitis, one of the major causes of late implant failure. In natural teeth, collagen fibers are firmly inserted and fixed in the cementum of the tooth and emerge perpendicular to the gingival tissue. In contrast, around dental implants, collagen fibers run predominantly parallel to the implant surface, allowing bacterial migration into the peri-implant interface that might lead to peri-implantitis. Previous studies have shown that nanostructured Ti surfaces improve gingival cell response in monolayer cell cultures. Here, we aimed at evaluating the implant–tissue interface using a 3D gingival tissue equivalent (GTE). First, we evaluated the GTE response to a nanostructured (NN) and machined Ti surface after the stimulation with *Porphyromonas gingivalis* lipopolysaccharide (LPS), to simulate peri-implantitis conditions. Thus, GTE viability, through MTT assay, the release of metalloproteinase-1 (MMP1) and its inhibitor (TIMP1) through ELISA, and the gene expression of extracellular matrix turnover genes by real-time RT-PCR were analyzed. Second, GTE–implant interaction was characterized by serial block face scanning electron microscopy, and collagen-1 orientation at the tissue–implant interface was analyzed by immunofluorescence. While a similar GTE response to LPS stimulation was found for both implant surfaces, a higher proportion of collagen oriented perpendicular to the implant was observed on the NN implant surface. Thus, our results indicate that the nanostructuration of titanium dental implant abutments could allow the correct orientation of collagen fibers and greater soft tissue sealing, while keeping biocompatibility levels and LPS response comparable.

## 1 Introduction

Since the introduction of the Branemark system for dental implants in 1971, the research on dental implants’ design and materials has increased.^1^ In spite of its high success rate, the absolute number of dental implant failure becomes significant and causes economic and social impact. In particular, the long-term clinical efficacy of titanium dental implants is influenced by peri-implantitis, an inflammatory reaction in the tissues surrounding an implant, which includes both soft tissue inflammation and progressive bone loss.^2^ Although there is some discrepancy in the reported data,^3^ recent studies have found that the peri-implantitis prevalence ranges between 0 and 39.7% depending on different case definitions.^4−6^

A good seal between the soft (gum) and hard (bone) tissues establishes a biological seal between the implant and oral cavity and drastically reduces the risk of peri-implantitis and implant failure.^7^ This biological seal protects the cells from bacterial penetration, avoiding gingival recession and bone resorption. Thus, a proper three-dimensional structure and function of the peri-implant soft tissue is a prerequisite for a long-term stable implant.

Studies with animal models have shown similarities and differences in the soft tissue attachment around natural teeth and dental implants. A key difference is the orientation of the collagen fibers; while in natural teeth these fibers are perpendicularly attached to the teeth cementum, in dental implants, they present a parallel circular arrangement. This fact, together with the lack of attachment structures such as hemidesmosomes, contribute to a weaker biological seal between the gingiva and the implant.^8−11^

Implant surface topography can modulate cell behavior by mechanotransduction. Thus, topographical features induce mechanical signals that are converted to biochemical signals, influencing the cell response to the surface.^12−15^ A nanoscale geometry can be achieved on Ti surfaces using different approaches, with electrochemical anodization being one of the most frequently used.^16^ In a previous study, we compared different nanostructures and selected a nanonet (NN) surface that resembled trabecular bone morphology at the nanoscale. This NN surface induced a higher frequency of alignment and a higher cell differentiation of both human gingival fibroblasts and human bone marrow mesenchymal stem cells using monolayer cell cultures.^17^

To evaluate the tissue–implant interface, either monolayer cell culture models or *in vivo* experiments with animals are most commonly used. While cell monolayers lack the extracellular matrix components losing cell-to-cell and cell-to-matrix interaction,^18,19^ the use of animals presents some ethical concerns. As an alternative, in vitro 3D tissue models are being developed, showing a higher degree of complexity and resembling more closely the in vivo situation.^18−21^ In fact, tissue-engineered oral mucosa models have been validated for cosmetic testing as an alternative to animal use.^22,23^

In this study, we aimed to better evaluate the tissue–implant interface and collagen orientation toward the implant using a three-dimensional gingival tissue equivalent (GTE) described by Dongari-Bagtzoglou and Kashleva^24^ and used in previous studies by our research team.^25−27^ The protocol was adapted to allow the development of this GTE around a titanium disc using two different implant surfaces: a machined implant and a nanostructured nanonet (NN) implant. We hypothesized that the NN surface would improve the implant–tissue interaction and induce a perpendicular collagen fiber orientation.

## 2 Experimental Section/Methods

### 2.1 Materials

Machined titanium discs, c.p. grade IV, 6.2 mm diameter, and 2 mm height were purchased from Implantmedia (Lloseta, Spain).

### 2.2 Surface Nanostructuration

Titanium discs were polished and cleaned as previously described.^28^ Afterward a nanonet (NN) nanostructure was produced using an Autolab electrochemistry instrument (Metrohm Autolab BV, Utrecht, The Netherlands), with the titanium samples as an anode and a platinum electrode (Metrohm Autolab BV, Utrecht, The Netherlands) as a cathode, as described in a previous study.^17^

### 2.3 Cell Culture

Immortalized Human Gingival Fibroblasts-hTERT (iHGF) (Applied Biological Materials Inc., Richmond, BC, Canada) and Immortalized Human Gingival Keratinocytes Gie-No3B11 (iHGK) (Applied Biological Materials Inc., Richmond, BC, Canada) were cultured as previously described.^25^ Cultures of each cell type with 70–80% confluence were used for the construction of GTE, as described in section 2.4.

### 2.4 Engineering 3D Gingival Tissue Equivalent (GTE)

**Figure 1:**
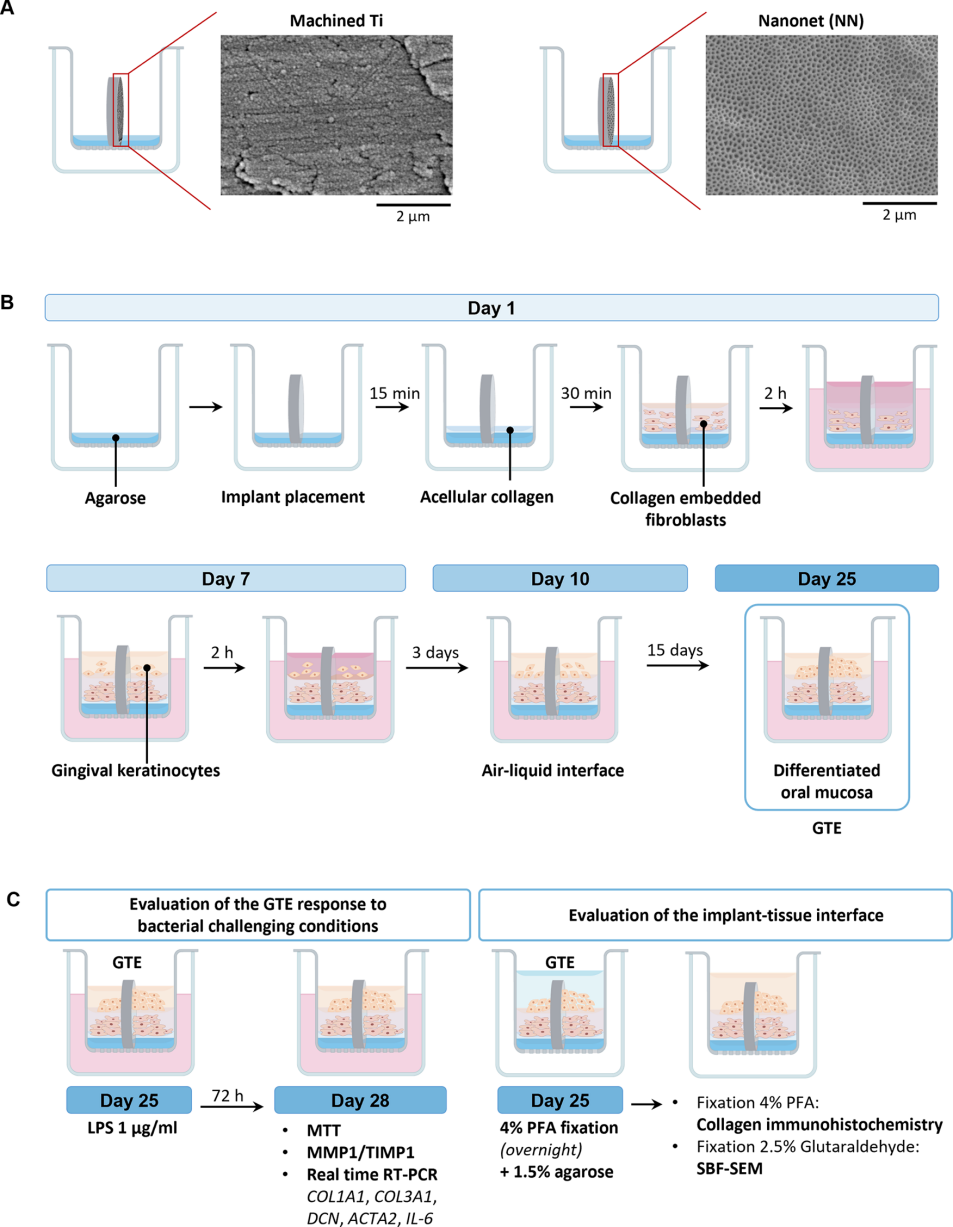
Experimental setup. (A) Representative scanning electron microscopy images of the two different surfaces used for the study, Ti and NN. (B) The figure represents the process followed to produce the 3D gingival tissue equivalent (GTE) around a Ti disc. (C) Schematic representation of the tests performed with the GTE.

The gingival tissue equivalent (GTE) was constructed as described by Dongari-Bagtzoglou and Kashleva^24^ and using the same protocol explained in previous publications from our research team^25−27^ with some modifications to allow the development of the GTE around Ti discs. First, 80 μL of 1.5% Agar prepared in DMEM low glucose 1% P/S were placed in each 24-well transwell insert with 0.4 μm pores (Sarstedt). Before solidifying, Ti discs were placed in the transwell inserts as shown in Figure 1 to avoid any movement of the disc during the further culturing process. Once the agar had solidified, a rat tail type I collagen solution (ThermoFisher Scientific, Waltham MA, USA) (2.2 mg/mL) was placed on each side of the implant and incubated for 30 min at room temperature. Then, the collagen solution was mixed with iHGF (10^5^ cells/well) and pipetted into the insets, on each side of the implant. This was incubated for 1 h at room temperature and 1 h at 37 °C, 5% CO_2_ before adding the fibroblasts cell culture medium. The fibroblast-embedded collagen was cultured at 37 °C and 5% CO_2_ for 7 days. After, iHGK (2.5 × 10^5^ cells/well) were added on top, and GTEs were cultured at 37 °C, 5% CO_2_ for 3 days submerged in keratinocyte medium. Then, GTEs were lifted to an air–liquid interface and incubated at 37 °C, 5% CO_2_ for 15–17 days in airlift culture medium (AL) prepared as previously explained.^25^ The AL medium was renewed every 2 days. After 25 days, GTEs were fixed for collagen immunohistochemistry or SBF-SEM analysis, or alternatively an inflammatory stimulus was applied, as indicated in section 2.5.

### 2.5 Evaluation of the GTE Response to Bacterial Challenging Conditions with Lipopolysaccharide

Lipopolysaccharide (LPS) from *Porphyromonas gingivalis* (Invivogen, San Diedo, CA, USA) was used to produce an inflammatory stimulus on the GTE in order to mimic a peri-implantitis situation. It was added to the tissue cultured with the different modified surfaces in a concentration of 1 μg/mL. After 72 h of incubation, several differentiation and inflammatory markers were analyzed to evaluate whether the surface modifications studied could alter the cell response to this stimulus.

### 2.6 Cell Viabilty Test (MTT)

At the end of the incubation period, with the LPS, an MTT assay to measure GTE viability was performed as previously described.^25^ Results are expressed as percentage of viability compared to the negative control. Four replicates from each group (*n* = 4) were used in this experiment.

### 2.7 MMP-1 and TIMP-1 Determinations by ELISA

Metalloproteinase-1 (MMP1) and its inhibitor (TIMP1) detection from GTE cell culture media after 72 h of inflammatory stimulus (LPS 1 μg/mL) was performed using commercially available ELISA kits according to the manufacturer instructions (Sigma, St. Louis, MO, USA). Eight replicates from each group (*n* = 8) were used in this experiment.

### 2.8 Gene Expression by RT-PCR

After 72 h of inflammatory stimulus (LPS 1 μg/mL), total RNA was isolated using tripure isolation reagent (Roche, Basel, Switzerland), according to the manufacturer’s protocol and quantified at 260 nm using a Nanodrop spectrophotometer (NanoDrop Technologies, Wilmington, DE, USA). cDNA synthesis and Real Time RT-PCR were performed as previously described.^25^ Three reference genes were used in the Real Time RT-PCR (glyceraldehyde-3-phosphate dehydrogenase (*GAPDH*), beta-actin (*ACTBL2*), and 18S rRNA (*18S rRNA*)) and several target genes were analyzed (Table 1). Seven replicates from the Ti group (n =7) and Eight replicates from the NN group (*n* = 8) were used in this experiment.

**Table 1:** Genes and Primers Used in Gene Expression Analysis^a^

Related function	Gen	Primer sequence (5′-3′)	Product size (bp)	genBank ID
ECM component	collagen I α1 (*COL1A1*)	S: CCTGACGCACGGCCAAGAGG A: GGCAGGGCTCGGGTTTCCAC	122	NM_000088.3
ECM component	collagen III α1 (*COL3A1*)	S: GGCCTACTGGGCCTGGTGGT A: CCACGTTCACCAGGGGCACC	190	NM_000090.3
ECM component	decorin (*DCN*)	S: ATCTCAGCTTTGAGGGCTCC A: GCCTCTCTGTTGAAACGGTC	146	NM_001920.3
wound healing/fibrogenic	alpha-smooth muscle actin 2 (*ACTA2*)	S: TAAGACGGGAATCCTGTGAAGC A: TGTCCCATTCCCACCATCAC	184	NM_001141945.1
proinflammatory cytokine	*IL6*	S: AGGAGACTTGCCTGGTGAAA A: GCATTTGTGGTTGGGTCAG	196	NM_000600.3
reference gene	glyceraldehyde-3-phosphate dehydrogenase (*GAPDH*)	S: TGC ACC ACC AAC TGC TTA GC A: AAG GGA CTT CCT GTA ACA A	87	NM_002046.3
reference gene	beta-actin (*ACTBL2*)	S: CTG GAA CGG TGA AGG TGA CA A: AAG GGA CTT CCT GTA ACA A	140	NM_001101.3
reference gene	18S rRNA (*18S rRNA*)	S GTAACCCGTTGAACCCCATT A: CCATCCAATCGGTAGTAGCG	151	NR_146156.1

a Sequence of sense (S) and antisense (A) primers was used in the real-time RT-PCR of reference and target genes. Base pairs (bp).

### 2.9 Sample Fixation for the Evaluation of the Implant–Tissue Interface by SBF-SEM

The samples were fixed in an aqueous solution of formaldehyde at 4% overnight at room temperature. Then, 100 μL of 1.5% agarose was added on top of each sample to secure the implant from moving, and they were fixed again with 2.5% glutaraldehyde for 1 h for SBF-SEM, or with 4% PFA for 1 h for collagen immunohistochemistry. Then, samples were kept in PBS at 4 °C until use.

### 2.10 Sample Staining, Resin Embedding, and Serial Block Face Scanning Electron Microscopy (SBF-SEM)

**Figure 2:**
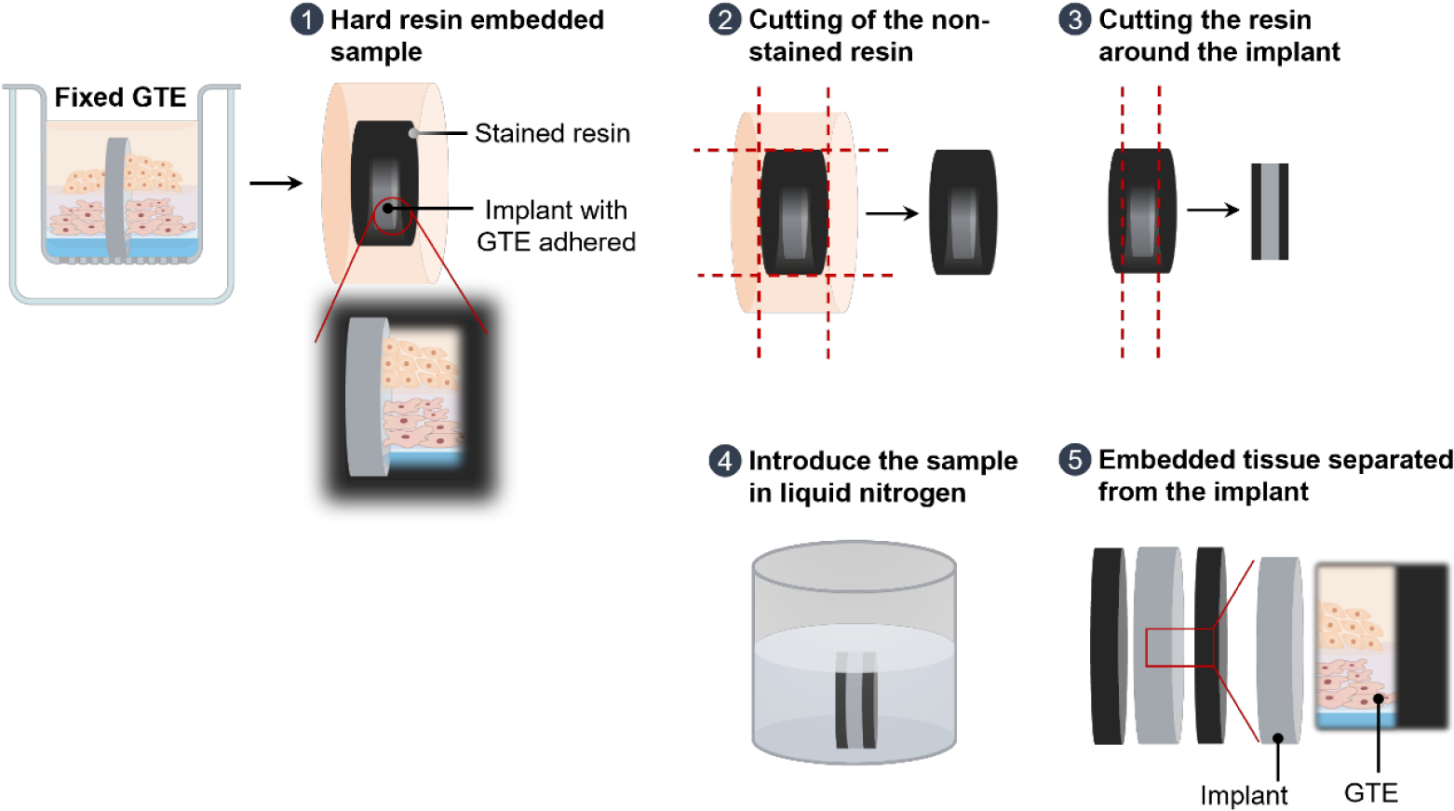
Sample preparation for SBF-SEM. Schematic representation of the process followed for the separation of GTE from the Ti disc.

Ti discs and the GTE constructs were taken out of the transwell insert and separated from the agarose before staining. Samples were washed with water and incubated for 1 h in 1% osmium tetroxide (Agar Scientific, Essex, UK) and 1.5% potassium ferrocyanide (Sigma-Aldrich, St. Louis, MO, USA) in cacodylate buffer 0.1M. Then, samples were washed again with distilled water 3 times. When the water was clear, samples were put in a 1% thiocarbohydrazide solution (ACROS Organics, Waltham, MA, USA) for 30–60 min at room temperature. Following this, samples were washed with distilled water several times, until all of the crystals formed were dissolved and were incubated for 30–60 min with a 1% osmium tetroxide solution (Agar Scientific, Essex, UK). After washing again with water, samples were incubated overnight in 1% uranyl acetate (Agar Scientific, Essex, UK) solution at 4 °C. Finally, samples were incubated for 30 min at 60 °C in a Walton’s Lead aspartate (Sigma Aldich, St. Louis, MO, USA) solution before dehydration and embedding in a 812 hard type resin (TAAB). Most resins for embedding are hydrophobic, and the sample water must be dehydrated with ascending ethanol (Thermo Fischer Scientific, Waltham, MA, USA), grades (30%, 50%, 70%, 90%, and 100%, for 15 min each), and exchanged by pure acetone (Thermo Fisher Scientific, Waltham, MA, USA) (twice, for 30 min each). After the dehydration process, samples were embedded in resin, allowing its hardening; liquid resin was infiltrated in the samples and polymerized without affecting the structure. Samples were placed in ascendent TAAB 812 hard resin acetone solutions (25%, 50%, 75%, and 100%), and then curated in TAAB 100% resin at 60 °C for at least 24 h. Next, in order to analyze the samples using SBF-SEM, it was necessary to separate the tissue from the Ti disc. First, the resin was cut around the Ti implant with a fretsaw, leaving two pieces of resin-embedded tissue linked by the implant. Then, it was introduced into liquid nitrogen for a few seconds, until the three pieces separated as illustrated in Figure 2.

Before the SBF-SEM analysis, samples were cut in small fragments and mounted on aluminum pins (Micro to Nano, Haarlem, Netherlands) with Permabond engineering adhesives superglue and trimmed with an ultramicrotome with a diamond knife. Then, samples were introduced into the SBF-SEM chamber and imaged to select the area of interest. After that area was selected, the sample was further trimmed to generate a block face of approximately 800 μm × 800 μm. Finally, samples were imaged in an FEI Quanta 250 FEG containing a Gatan 3view. The microscope was set to 3.8 kV with 0.45 Torr chamber pressure. A series of images was collected with a cut depth of 50 nm and a pixel size of 10 nm.

The SBF-SEM analysis uses a microtome that sits in the chamber of a SEM. The top of the sample block face is imaged before it is cut by the microtome, revealing a new block face that is imaged again. This process is repeated until the desired depth of the sample has been analyzed.

### 2.11 Three-Dimensional Reconstruction of the Samples

The data obtained from SBF-SEM analysis was binned in Z, contrast inverted, and flipped in X using IMOD (version 4.11). Then, 3D Slicer’s volume rendering program was used to generate the three-dimensional renderings of the samples.

### 2.12 Collagen Immunostaining

The Ti discs and GTE constructs were taken out of the transwell insert by pullout and transferred to a 24 well-plate with the modified face of the implant facing up. Then, samples were permeabilized with PBS-Triton 0.5% for 1 h. Then, GTEs were blocked with bovine serum albumin (5%, 1 h; Sigma-Aldrich, St. Louis, MO, USA), followed by incubation with 4 μg/mL of anticollagen recombinant rabbit monoclonal antibody (Invitrogen) for 1 h, and then labeled with 5 μg/mL of Alexa Fluor 488 goat antirabbit IgG secondary antibody (Thermo Scientific, Rockford, IL, USA) for 1 h. Samples were then mounted with DAPI-Fluoroshield (Sigma-Aldrich, St. Louis, MO, USA) and visualized under a confocal microscope. Images were analyzed using ImageJ software (National Institutes of Health, Bethesda, MD, USA). A skeletonization algorithm was applied to the images,^29^ and then they were analyzed using the FIJI software Plugin OrientationJ^30,31^ (National Institutes of Health, Bethesda, MD, USA).

### 2.13 Statistical Analysis

All data are presented as mean values ± standard deviation (SD). Shapiro–Wilk test was done to assume parametric or nonparametric distributions. Variance homogeneity was analyzed using the Levene test. Parametric data were analyzed by Student’s *t* test. Nonparametric data were analyzed by Mann–Whitney. Results were considered statistically significant at *p* < 0.05. SPSS program for Windows (version 17.0, SPSS Inc., Chicago, IL, USA) and GraphPad Prism (version 7, La Jolla, CA, USA) were used.

## 3 Results and Discussion

### 3.1 Evaluation of the GTE Response to Bacterial Challenging Conditions

Peri-implantitis is mainly caused by bacterial infection and the host response to the bacterial challenge. *P. gingivalis* is one of the principal pathogens of human periodontitis,^32,33^ eliciting its virulence in part through lipopolysaccharide (LPS) release. LPS stimulates the expression of inflammatory cytokines and chemokines in the host tissue, which ends in tissue breakdown.^34^

**Figure 3:**
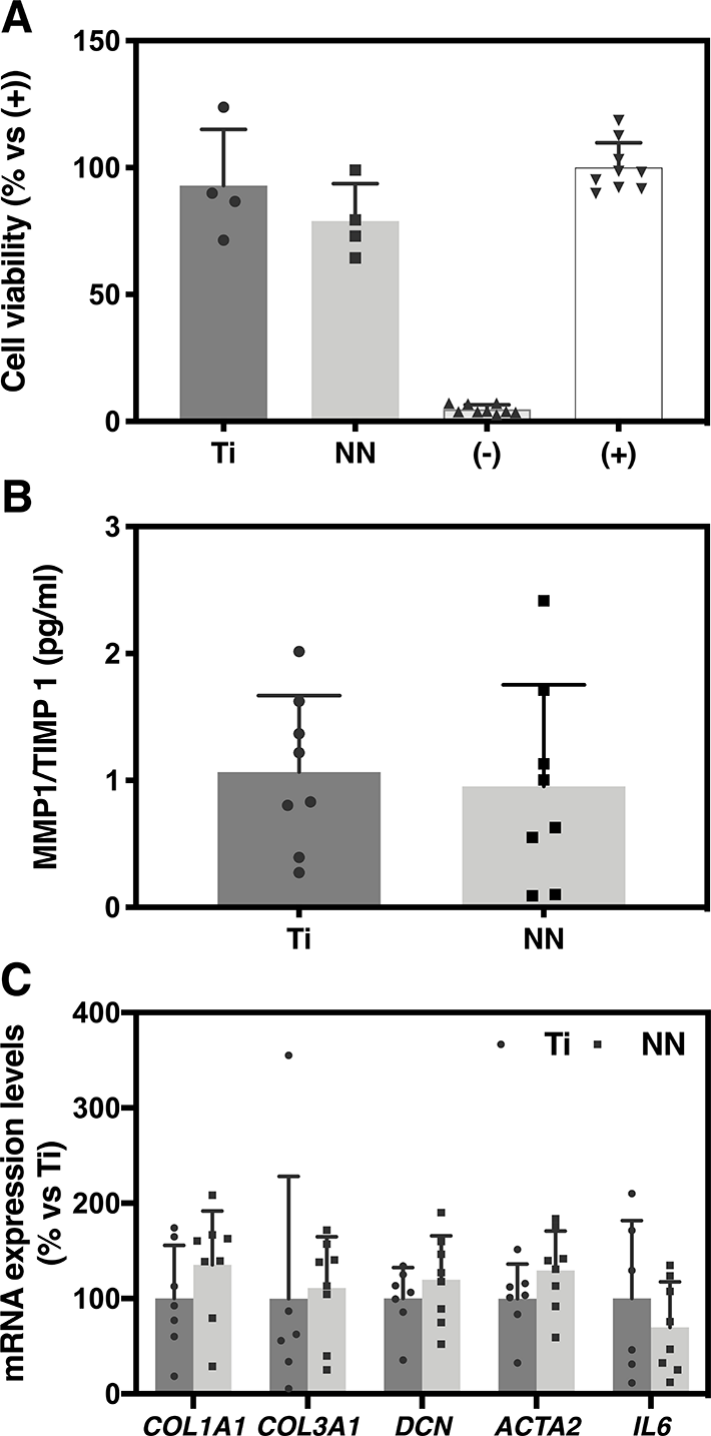
GTE response to different surfaces after an inflammatory stimulus. (A) Viability of gingival tissue equivalent 72 h after the *Porphyromonas gingivalis* LPS stimulus measured with the MTT test. Positive control was obtained from the culture media of GTE treated with PBS and set at 100%. Negative control was obtained from culture media of GTE treated with 5% SDS diluted in PBS (1:1). Values represent the mean ± SD (*n* = 4; three independent experiments were performed). (B) MMP1 and TIMP1 release by GTE after LPS stimulation for 72 h measured by ELISA. Values represent the mean ± SD (*n* = 8; three independent experiments were performed). (C) GTE mRNA expression levels of *COL1A1*, *COL3A1*, *DCN*, *ACTA2*, and *IL6* after LPS stimulation for 72 h. Values represent the mean ± SD (*n* = 7 for Ti and *n* = 8 for NN; three independent experiments were performed). Results were statistically compared by Student’s *t* test for parametric data, and by Mann–Whitney for nonparametric data (*COL3A1* mRNA expression levels). Nonsignificant differences were found.

Here, in order to evaluate the effect of nanostructuration on tissue response to a bacterial challenge, gingival tissue equivalents grown around implants were stimulated with LPS from *P. gingivalis* (Figure 3). As shown in Figure 3A, the two different implant surfaces were biocompatible with all tissues presenting high levels of viability. However, in previous studies performed in monolayer cell cultures, an increased cell viability in the NN surface was demonstrated.^17,35^ This result confirms the biocompatibility of the nanostructured surface in a more complex 3D model, which can resemble more closely the in vivo situation.

No effect of surface nanostructuration was found on matrix metalloproteinase-1 (MMP1) production or on its inhibitor (TIMP-1) (Figure 3B). MMP-1 regulates collagen degradation, and its inhibitor TIMP-1 controls its activity through proteolysis, to regulate the extracellular matrix turnover.^36,37^ It has been described that under inflammatory conditions the MMP/TIMP ratio is upregulated, all together boosting collagen degradation.^38^

With regards to gene expression analysis, although no statistically significant differences were found, we could observe a tendency for increased expression levels of cell matrix turnover related genes (*COL1A1*, *COL3A1*, *DCN*, *ACTA2*) and decreased expression levels of the pro-inflammatory cytokine IL6 for the nanostructured surface compared to the control (Figure 3C). A higher production of collagen type I and III is associated with a higher gingival differentiation, associated with better wound healing around a dental implant.^39^ DCN is a small proteoglycan highly expressed in human gingiva that regulates collagen fibril organization, including collagen type I and III, the major protein components of gingival tissue extracellular matrix.^40^ ACTA-2 is a contractile protein that contributes to tissue repair during wound healing, but if it is overexpressed can lead to fibrogenic conditions.^41^ IL6 is considered a pro-inflammatory cytokine, that can induce bone loss, and its increase has been related in several studies with peri-implantitis.^42^ Our results could indicate a higher tissue integration with this nanostructured surface, and the lack of statistical significance could be related to the fact that all the tissue was used for the gene expression analysis, and only a small part of the tissue is in direct contact with the surface. Previous studies with NN surfaces showed a higher cell differentiation for gingival fibroblasts and bone marrow mesenchymal stem cells. Specifically, gingival fibroblasts presented a higher collagen deposition when cultured on NN surfaces compared to Ti.^17^ However, that study was performed with monolayer cell cultures, where all the cells were in direct contact with the implant surface, while in the present study, gene expression was performed using all the GTE, where only the cells at the interface are in direct contact with the implant surface. Thus, future studies could pull out the GTE from the surface and analyze gene expression directly at the implant interface.

### 3.2 Evaluation of the Implant–Tissue Interface

Soft tissue integration (STI) establishes an effective biological seal between the oral cavity and implant. This integration at the dental implant abutments protects bone and implant from bacterial penetration, avoiding gingival recession and inflammation-driven bone resorption,^43^ and inhibits epithelial downgrowth.^44^ Thus, proper 3D structure and function of the soft tissue seal around dental implants is considered to be a prerequisite for achieving long-term stable peri-implant conditions.^45^

**Figure 4:**
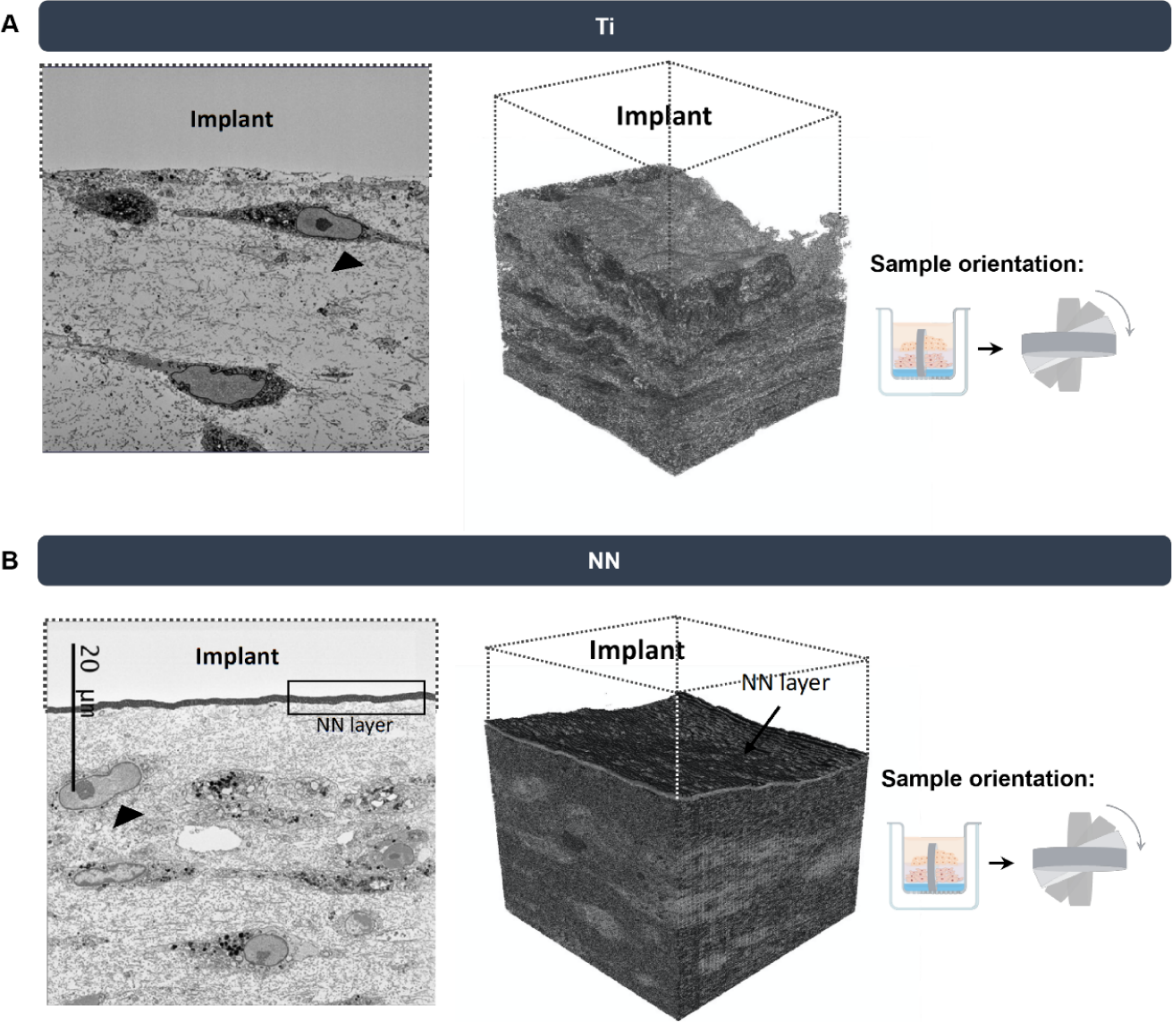
Representative images and 3D reconstructions of the GTE interface with Ti or NN implants acquired with SBF-SEM. (A) GTE interface and 3D reconstruction with the Ti implant (removed). (B) GTE interface and 3D reconstruction with the NN implant (removed, the nanostructure layer can be observed attached to the top side of the GTE, marked). Black arrows show the presence of gingival fibroblasts embedded in the collagen matrix. Scale bar showed in the image represents 20 μm.

In order to evaluate tissue–implant integration, we used SBF-SEM for the tissue–implant interface examination. We were able to create 3D representations of the gingival tissue equivalents developed around the different implant surfaces (Ti and NN). To do so, we adapted the staining and inclusion protocol to this type of sample and separated the implant from the tissue. Although the separation of the 3D GTE and the implant can affect the implant–tissue interface, the fact that the NN layer is still attached to the 3D GTE after separation (Figure 4B) could indicate that the technique used for the implant detachment creates a clean separation that does not alter the interface structure. In a previous study, the interface between Ti implants and an engineered oral mucosa was evaluated by using a focused ion beam (FIB) without the need to separate the Ti implant. However, using FIB the sample structure can be altered, impairing the study of the implant–tissue interface.^46^ Inside the collagen matrix (Figure 4A,B), several gingival fibroblasts could be observed, showing a healthy elongated morphology with a well-defined cell nucleus and organelles (marked with a black arrow) for both implant groups. In addition, when separating the embedded tissue from the implant for SBF-SEM evaluation (Figure 2), we observed that the nanostructure layer came out of the tissue (Figure 4B). A similar phenomenon has been observed before, where a layer of TiO_2_ was found adhered to the tissue-engineered oral mucosa after its separation from the bulk implant.^46^ Hence, further studies are needed to test the safety of these nanostructures before their clinical translation.

The soft tissue–implant interface has been described as connective tissue resembling the scar tissue with collagen fibers running predominantly parallel to the implant surface and not attached to it.^11,47−50^ In contrast, in the natural tooth, these collagen fibers are firmly inserted and fixed in the cementum of the tooth and emerge perpendicularly into the gingival tissue. This inferior attachment of the gingiva around implants compared to the physiological situation is assumed to be one important reason for bacterial migration into the peri-implant interface^51^ and subsequently for development of peri-implantitis.

Here, we hypothesized that the NN pores created on the implant surface might serve as a means for the collagen fibers to insert into them and grow perpendicularly from the nanostructured surface into the gingival tissue. In order to confirm our hypothesis, immunofluorescence staining of the collagen was performed on the tissues attached to the different surfaces after pulling the implant out of the GTE.

**Figure 5:**
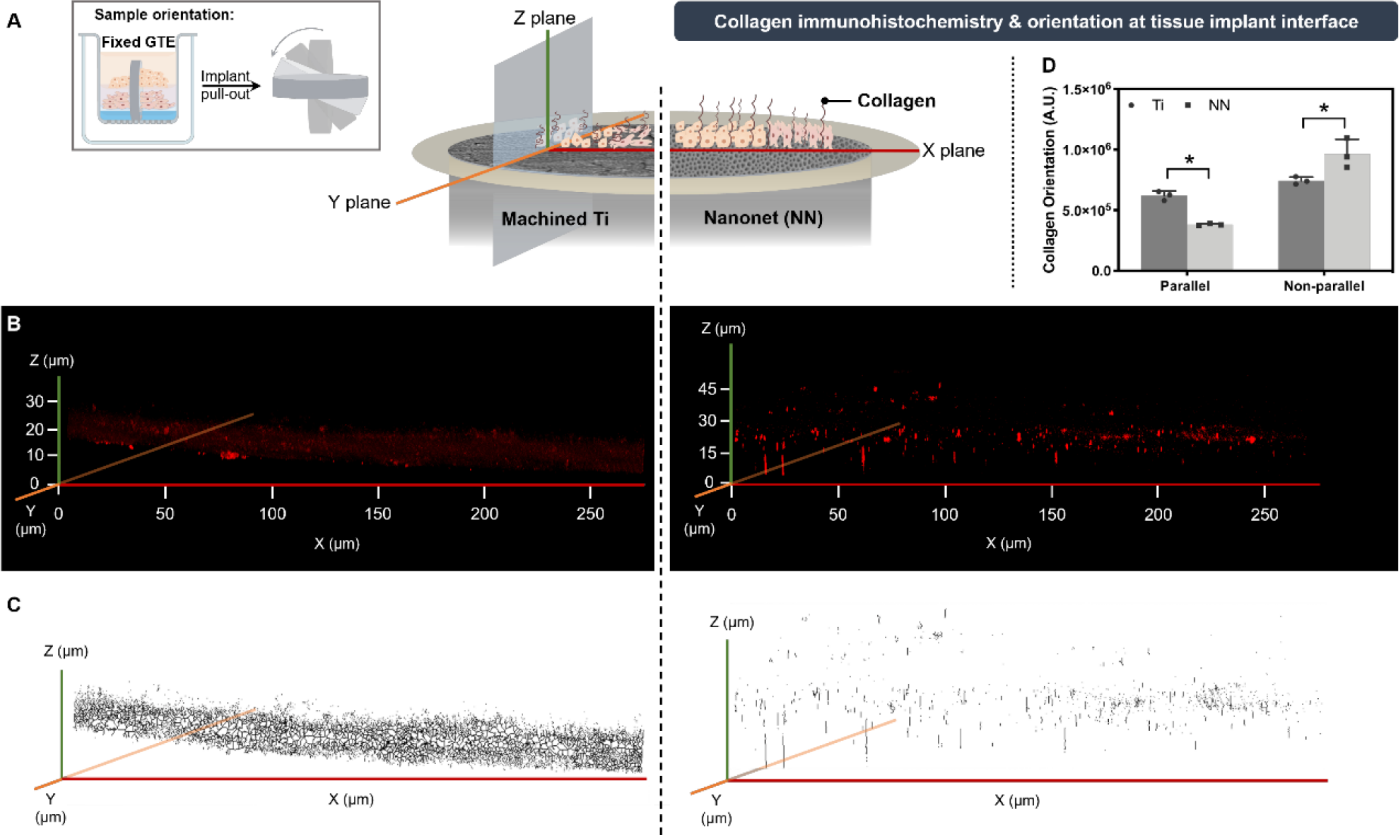
(A) Collagen-1 immunohistochemistry three-dimensional reconstruction along the *z*-axis of the GTE-implant interface. (B) Collagen skeleton reconstruction after applying the skeletonization algorithm. (C) Schematic representation of the implant pull out and evaluation of the immunostained collagen. (D) Collagen fiber orientation to the implant surface. The skeletonization algorithm was applied to confocal images and scores for each orientation degree were obtained through the FIJI software Plugin OrientationJ. Then, we considered as parallel orientation the addition of the scores obtained from the skeleton reconstruction from −30° to +30°, and perpendicular orientation all the rest (−90° to −30° and +30° to +90°). A.U (Arbitrary Units). Values represent the mean ± SD (*n* = 3). Results were statistically compared by Student’s *t* test: *p < 0.05 versus Ti.

Figure 5 shows a 3D reconstruction of the *z*-axis of collagen immunohistochemistry of the GTE-implant interface performed as indicated in Figure 5A. A skeletonization algorithm was applied to the images obtaining the reconstruction showed in Figure 5C, which represents the collagen fiber orientation on the GTE. According to the results observed in Figure 5, we can confirm an effect of nanostructuration on the collagen orientation on the tissue–implant interface. Both, fluorescent images (Figure 5B) and the collagen skeleton reconstruction (Figure 5C) show increased parallel orientation of collagen along the z-plane in control Ti implants compared to the perpendicular ones observed in NN implants. This observation was quantified (Figure 5D), confirming the significantly higher amount of collagen oriented perpendicularly to the implant on the NN structures versus the higher parallel orientation on control Ti surfaces. This could indicate a perpendicular integration of the collagen fibers into the NN structure, or at least that the nanostructure features might induce the collagen fibers to orient perpendicularly to the implant surface. This is the first report showing this rearrangement of collagen fibers induced by nanostructured surfaces as far as we are concerned. In a previous study, NN surfaces induced an oriented alignment of the human gingival fibroblasts and human bone marrow mesenchymal stem cells, which was not observed in the non-nanostructured discs. This cell orientation correlated with a higher cell differentiation, which in human gingival fibroblasts, resulted in a higher collagen deposition.^17^ Here, the use of a GTE instead of cell monolayers has allowed a better assessment of the implant–tissue interface, and to have a more structured extracellular matrix, allowing us the assessment of collagen orientation.

Although further in vivo evidence is needed to confirm our hypothesis, the results obtained so far indicate that nanostructuration may allow tissue sealing around the implant more similar to the natural tooth, with collagen fibers inserted and fixed into the implant and emerging perpendicularly into the gingival tissue.

## 4 Conclusion

Nanostructuration of Ti produced biocompatible implant surfaces with a similar tissue-response to LPS stimulation compared with machined implants. More remarkably, nanostructuration induced a higher proportion of collagen orientation perpendicular to the implant, resembling the natural situation in which collagen emerges perpendicularly from the cementum of the tooth into the gingival tissue. This tissue–implant interaction could allow a better soft tissue sealing around dental implants and, in turn, prevent peri-implantitis.
